# Structural characterization and in vitro lipid binding studies of non-specific lipid transfer protein 1 (nsLTP1) from fennel (*Foeniculum vulgare*) seeds

**DOI:** 10.1038/s41598-020-77278-6

**Published:** 2020-12-04

**Authors:** Mekdes Megeressa, Bushra Siraj, Shamshad Zarina, Aftab Ahmed

**Affiliations:** 1 Biomedical and Pharmaceutical Sciences, Chapman University School of Pharmacy, 9401 Jeronimo Road, Irvine, CA 92618 USA; 2 Dr. Zafar H. Zaidi Center for Proteomics, University of Karachi, Karachi, 75270 Pakistan

**Keywords:** Biochemistry, Biological techniques, Computational biology and bioinformatics, Plant sciences

## Abstract

Non-specific lipid transfer proteins (nsLTPs) are cationic proteins involved in intracellular lipid shuttling in growth and reproduction, as well as in defense against pathogenic microbes. Even though the primary and spatial structures of some nsLTPs from different plants indicate their similar features, they exhibit distinct lipid-binding specificities signifying their various biological roles that dictate further structural study. The present study determined the complete amino acid sequence, in silico 3D structure modeling, and the antiproliferative activity of nsLTP1 from fennel (*Foeniculum vulgare*) seeds. Fennel is a member of the family Umbelliferae (Apiaceae) native to southern Europe and the Mediterranean region. It is used as a spice medicine and fresh vegetable. Fennel nsLTP1 was purified using the combination of gel filtration and reverse-phase high-performance liquid chromatography (RP-HPLC). Its homogeneity was determined by sodium dodecyl sulfate polyacrylamide gel electrophoresis (SDS-PAGE) and matrix-assisted laser desorption/ionization-time of flight (MALDI-TOF) mass spectrometry. The purified nsLTP1 was treated with 4-vinyl pyridine, and the modified protein was then digested with trypsin. The complete amino acid sequence of nsLTP1 established by intact protein sequence up to 28 residues, overlapping tryptic peptides, and cyanogen bromide (CNBr) peptides. Hence, it is confirmed that fennel nsLTP1 is a 9433 Da single polypeptide chain consisting of 91 amino acids with eight conserved cysteines. Moreover, the 3D structure is predicted to have four α-helices interlinked by three loops and a long C-terminal tail. The lipid-binding property of fennel nsLTP1 is examined in vitro using fluorescent 2-p-toluidinonaphthalene-6-sulfonate (TNS) and validated using a molecular docking study with AutoDock Vina. Both of the binding studies confirmed the order of binding efficiency among the four studied fatty acids linoleic acid > linolenic acid > Stearic acid > Palmitic acid. A preliminary screening of fennel nsLTP1 suppressed the growth of MCF-7 human breast cancer cells in a dose-dependent manner with an IC_50_ value of 6.98 µM after 48 h treatment.

## Introduction

Within the membranes of plant cell organelles, lipids go through metabolic activities, including anabolism, catabolism, and renewal^1^^.^ These organelles, however, do not have enzymes that are engaged to synthesize lipids. This necessitates an intracellular non-vesicular route for lipid mobilization that facilitates their movement inside the aqueous environment of the cytoplasm. Thus, a closer examination of lipid trafficking mechanisms had led to the identification of LTPs from spinach leaves for the first time in 1984 by Kader et al.^1–3^. These proteins exhibited the ability to transfer hydrophobic molecules, including phosphatidylcholine, phosphatidylglycerol, phosphatidylethanolamine, and phosphatidylinositol, from liposomes to mitochondria; bolstering the hypothesis that phospholipids are imported to intracellular organelle from outside through a transfer process by LTPs^2^. The proteins were named first as “phospholipid-exchange proteins” (PLEPs). Still, since there was no true one-for-one exchange of phospholipids noted between acceptor and donor membranes, the name “phospholipid-transfer protein” was given. Nonetheless, the generic name “lipid-transfer protein” was provided since it promotes the transfer of lipids other than phospholipids^4–6^. Consequently, “nonspecific lipid-transfer protein” was used as they lack specificity for the different phospholipids^7^. Since then, several nsLTPs have been purified and characterized from several plants species such as wheat (*Triticum aestivum*)^8^, Chinese cabbage (*Brassica rapa*)^9^, cumin (*Cuminum cyminum*)^10^, maize (*Zea mays*)^11^, castor bean (*Ricinus communis*)^12^, barley (*Hordeum vulgare*)^13^, Chinese lily (*Narcissus tazetta*)^14^, wild carrots (*Daucus carota*)^15^, eggplant (*Solanum melongena*)^16^ and ajwain *(Trachyspermum ammi*)^17^.

Plant nsLTPs are highly conserved small molecular weight proteins with a high isoelectric point (*pI*) between 9 and 11. Based on their sizes, they were classified into two subfamilies as nsLTP1 and nsLTP2 (9 kDa and 7 kDa), respectively^7,18^. Nevertheless, the newer classification method further grouped nsLTPs into nine types on the basis of amino acid sequence similarity and intervals of eight cysteine residues as the former method excludes the newly found anther-specific proteins exhibiting homology to plant nsLTPs. Later, with the advancement of technology, the 3D structures of nsLTPs have been resolved from different plants using Nuclear Magnetic Resonance spectroscopy (NMR), Infrared spectroscopy (IR), and X-ray crystallography^10,19,20^. Subsequently, it is reported that there exist eight cysteine residues at the conserved positions that form four disulfide bridges as well as four α-helices in nsLTP1s and three α-helices in nsLTP2s^18^, with a long C-terminus. The helices are situated in a folded manner creating an internal hydrophobic cavity suitable for the binding of lipids and other hydrophobic molecules^19^.

Most nsLTPs display broader lipid binding specificity *in vitr*o that they form non-covalent complexes with fatty acids, fatty acyl-CoA, hydroxylated fatty acids, phospholipids, and glycolipids. Some of them are also shown to accommodate large molecular weight hydrophobic molecules like prostaglandin B2 like wheat (*Triticum aestivum*) nsLTP^21^. Their binding affinities to various ligands are also noted to be variable depending on their 3D structures. Several nsLTPs bind to more than one lipid molecules at a time, while some do not bind at all due to steric hindrance of the bulky side chains of aromatic amino acids that made the hydrophobic cavity^22^. Among the lipids examined in binding studies, saturated lipids containing 16–18 carbons interact significantly compared to those with 12–14 or 20–22 carbons^23^. The presence of long carbon chains, hydroxyl group, one or two double bonds in the acyl chain, and *cis* configuration of the lipid molecules are determining factors that control their affinity to nsLTPs, as evidenced from a computational study on Asian rice (*Oryza sativa*)^24^.

NsLTPs are synthesized as preproteins in plant cells with a signal peptide at the N-terminal that is 21–27 amino acid residues in LTP1s and 27–35 residues in LTP2s, where they are secreted into apoplastic space^18,25^. Plant nsLTPs play a significant role beyond lipid shuttling between membranes. Instead, some of them are involved in growth and reproduction, symbiosis, defense against pathogenic microbes, and adaptation of plants to stress^22,25^. Their involvement in various active roles in plants' survival and their stability against denaturants, heat, and proteases, nsLTP are drawing research interests as drug carriers for targeted drug delivery and potential drugs to the existing deadly diseases^26,27^.

Therefore, this project focuses on nsLTP1 isolated from the seeds of fennel (*Foeniculum vulgare*), a member of the Umbelliferae (Apiaceae) plant family. It is an erect perennial-umbelliferous plant with hollow stems of length up to 2.5 m^28^. Fennel is among the most important medicinal plants that are native to southern Europe and the Mediterranean region. Ethnomedical uses involve cough, abdominal problems, eye diseases, fever, insomnia, kidney problems, mouth ulcer, and to enhance milk supply^29–31^. In support of its ethnomedical use, studies have shown the pharmacological activity of fennel, including antitumor, antibacterial, antifungal, antioxidant, antithrombotic, anti-inflammatory, oestrogenic, hepatoprotective, and antidiabetic^29,31–34^. Numerous pharmacological activities of fennel studied so far are based on aqueous and/organic extracts and essential oils, leaving proteins and peptides isolated unexplored. Hence, this project aims to isolate and characterize the primary structure of fennel nsLTP1 using N-terminal amino acid sequencing and 3D structural modeling using bioinformatics tools. The study further examines the in vitro lipid binding potential of fennel nsLTP1 using the fluorescent method and in vitro cytotoxic effects on the MCF-7 breast cancer cell line.

## Results

### Purification of nsLTP1

**Figure 1:**
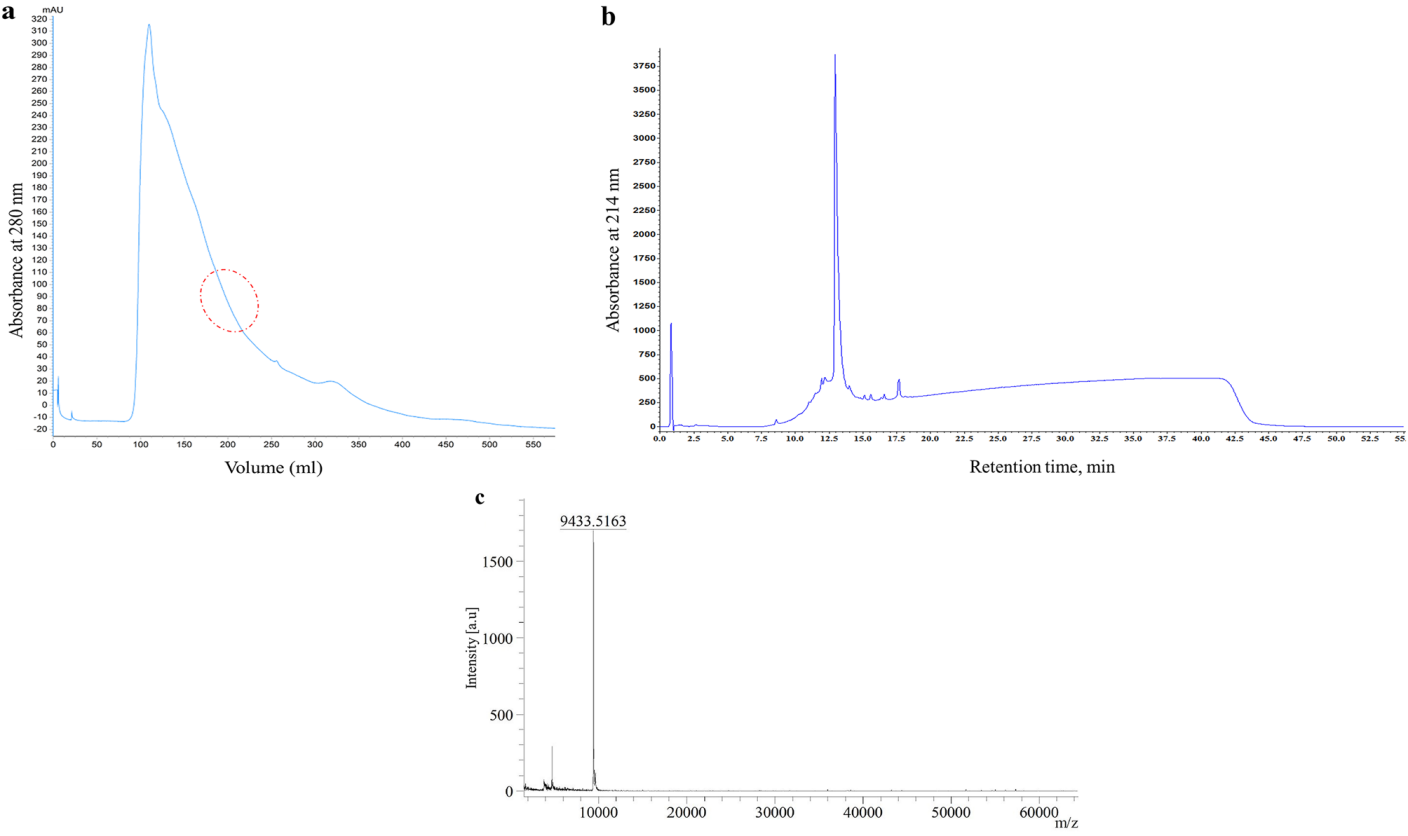
Chromatographic profile and mass spectrometric analysis of fennel nsLTP1. **(a**) Protein elution profile of crude protein sample precipitated from fennel (*Foeniculum vulgare*) seeds on HiPrep Sephacryl S-200 HR16/20 gel filtration column. The red-colored circle signifies the fractions containing nsLTP1. (**b**) 2D-RP-HPLC elution profile of pooled gel filtration fractions 39–43 comprising fennel nsLTP1 on Aeris Widepore 3.6UXB-C18 (150 × 2.1 mm) column; (**c**) MALDI-TOF mass spectra of RP-HPLC purified fennel nsLTP1.

The crude protein mixture was extracted successfully from defatted fennel seed in 20 mM Tris/HCl pH 8.0 at 4 °C and allowed to precipitate using 60% ammonium sulfate. The partially purified proteins recovered by gel filtration chromatography is indicated in (Fig. 1a). The fractions 39–43 were observed to have a protein band around 9–10 kDa by 12% Tris/Tricine SDS-PAGE gel electrophoresis (Supplementary Fig. S1). The pooled fractions were then subjected to RP-HPLC, which resulted in a well-defined peak eluted at a retention time of 13 min (Fig. 1b). Analysis of purified nsLTP1 on MALDI-TOF mass spectrometry depicted the mass at *m/z* 9433.5163 Da (Fig. 1c).

### Amino acid sequence

**Figure 2:**
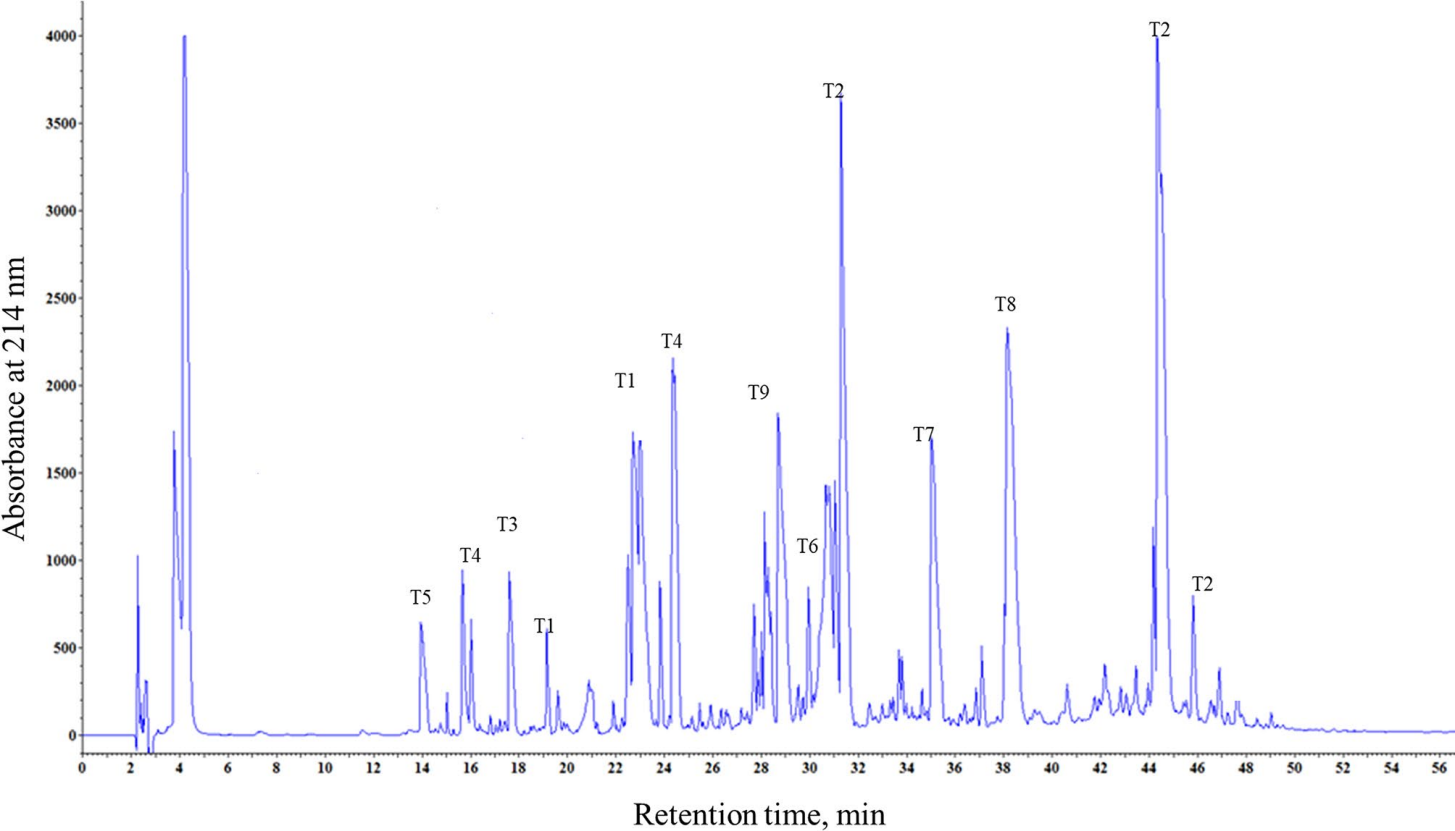
Peptide mass fingerprinting of nsLTP1 from fennel (*Foeniculum vulgare*) seeds. RP-HPLC chromatogram of tryptic digest of Cys-modified fennel nsLTP1 on Aeris widepore 3.6UXB-C18 (150 × 2.1 mm) column; The gradient from 0 to 60% acetonitrile in 55 min; flow rate 1 ml/min; absorbance monitored at 214 nm.

**Figure 3:**
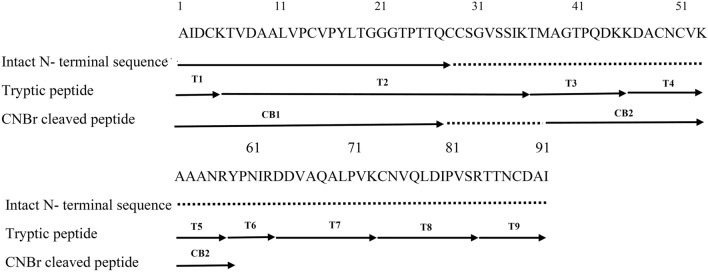
Amino acid sequence of nsLTP from fennel (*Foeniculum Vulgare*) seeds. Solid lines with T and CB represent peptides sequenced after trypsin and cyanogen bromide digestion, respectively.

The partial N-terminal amino acid sequence of purified nsLTP1 protein was identified using Edman sequencing (AIDCKTVDAALVPCVPYLTGGGTPT), which showed its homology to the nsLTP1 subfamily of proteins. Then, for accurate, complete primary structural determination, the protein chain was digested with TPCK (L-1-Tosylamide-2-phenylethyl chloromethyl ketone) treated trypsin. The RP-HPLC tryptic peptides elution profile is shown as (Fig. 2). The amino acid sequences of tryptic peptides acquired marked from T1 to T9 (numbered based on their position with respect to amino acid terminus) are determined. The orders of the tryptic peptides in the fennel nsLTP1 were achieved based on homology with other plant nsLTPs. Since peptide T9 lacks arginine and lysine residues, it was assumed that this is a carboxy-terminal end of the protein. Furthermore, CNBr cleavage digestion gave two large peptides CB1and CB2 confirming the presence of a single Met residue (Fig. 3). The protein region 38–51 was elucidated, and the presence of two lysine residues at positions 45–46 was confirmed by aligning the CB2 and T2/T3 peptides. The compilation of sequence data from the intact protein as well as from peptides generated from trypsin and CNBr digestion confirmed the complete primary structure of fennel nsLTP1 containing a total of 91 amino acids, including eight cysteine residues. These residues are conserved in the fennel nsLTP, thus enabling the same 3D structure known for a number of nsLTPs. The fully sequenced protein is deposited in the public protein database UniProt KnowledgeBase (UniProtKB) and assigned the accession number C0HLP9.

### 3D Structural modeling

**Figure 4:**
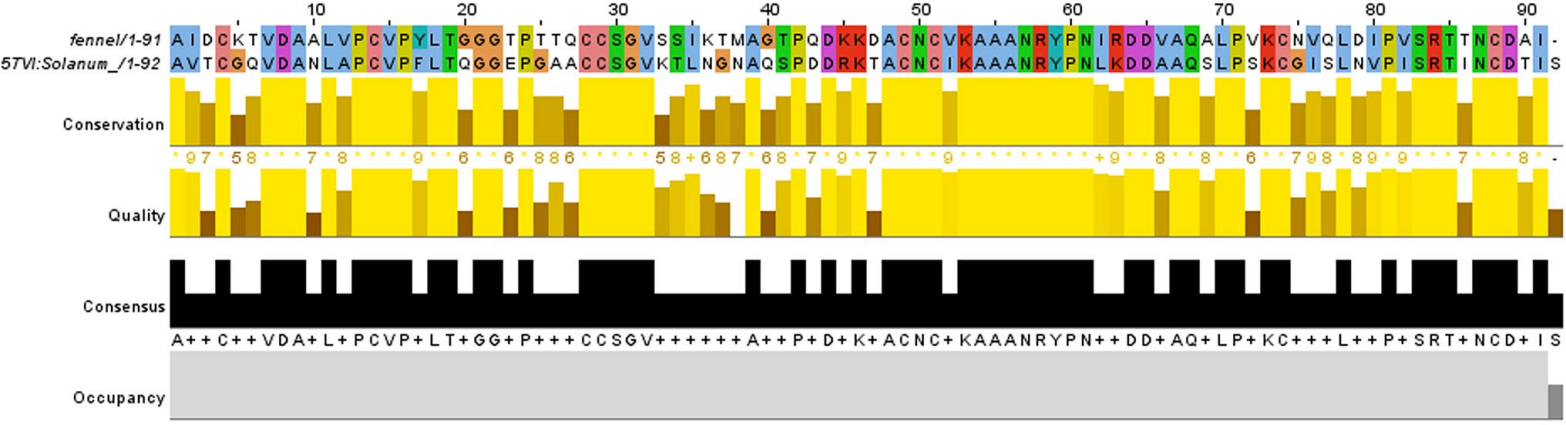
Pairwise sequence alignment of nsLTP1 from fennel (*Foeniculum vulgare*) and Eggplant (*Solanum melongena)* nsLTP1 through Clustal Omega as visualized by Jalview.

**Figure 5:**
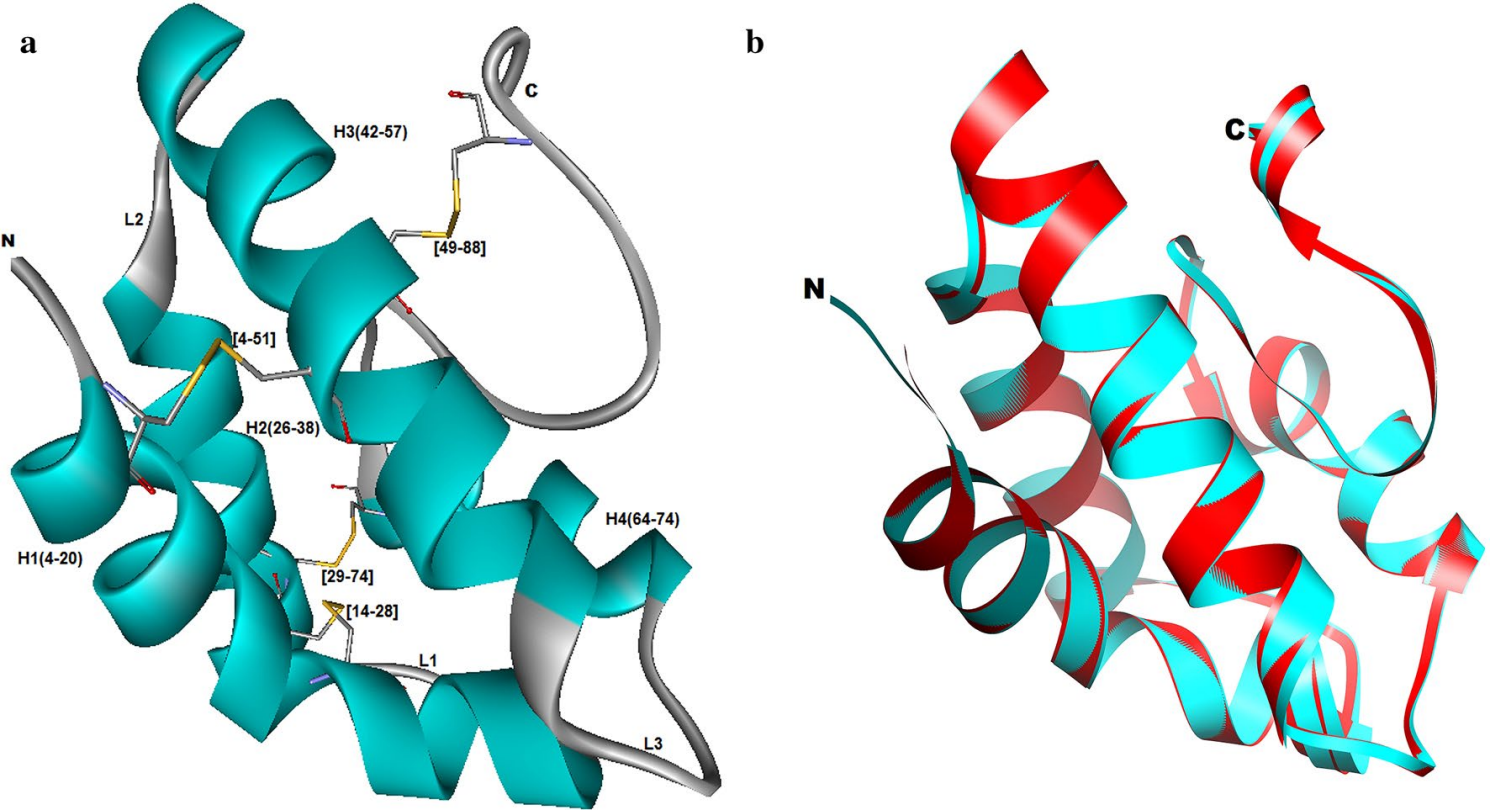
Spatial structure of nsLTP1 from fennel (*Foeniculum vulgare*) seeds and its comparison with template structure. (**a**) Modeled structure of nsLTP1 with ribbon representation. Helices (H1, H2, H3, and H4) are in blue. Loops (L1, L2, L3) are in grey. Disulfide bonds are in yellow. (**b**) Superimposition of modeled fennel nsLTP1 model (blue) with the template Eggplant (*Solanum melongena)* nsLTP1 (5TVI) (red).

Three-dimensional structure prediction of fennel nsLTP1 was conducted using bioinformatics tools. The BLASTp analysis of fennel nsLTP1 against PDB revealed potential templates (Supplementary Table S1), which were aligned with the target sequence (Supplementary Fig. S2). Of these, crystal structure coordinates of non-specific lipid transfer protein 5TVI_V from *S*. *melongena* (Eggplant) was taken based on its highest identity (59.34%) and similarity (74%) with the least E-value. Sequence alignment between the target (fennel nsLTP1) and template (*Solanum*) is shown in Fig. 4. The Modeller program was used to build the model of nsLTP1 from the selected template. The best model with the least energy assessed by DOPE score was selected and shown in Fig. 5a. The superimposition of the modeled structure with the template using Chimera gave RMSD 0.118 Å, suggesting a good fit (Fig. 5b). The quality of the constructed model was assessed by Ramachandran plot using PROCHECK (Supplementary Fig. S3), which showed that 93.5% of the amino acids were found in the most favorable allowed region, 5.2% in sterically allowed, and only 1.3% residues in disallowed regions. The energy profile of the model was evaluated by the PROSA tool, which gave a z-score − 5.4, suggesting it to be a good model. The modeled structure is assigned the accession number PM0082624 in the online protein model database (PMDB)^35^. Fennel nsLTP1 has 91 residues whereas 3D structure is predicted to have four α-helices that are interlinked by a total of three loops L1, L2, and L3; as well as a long C-terminal tail.

### Molecular docking

**Figure 6:**
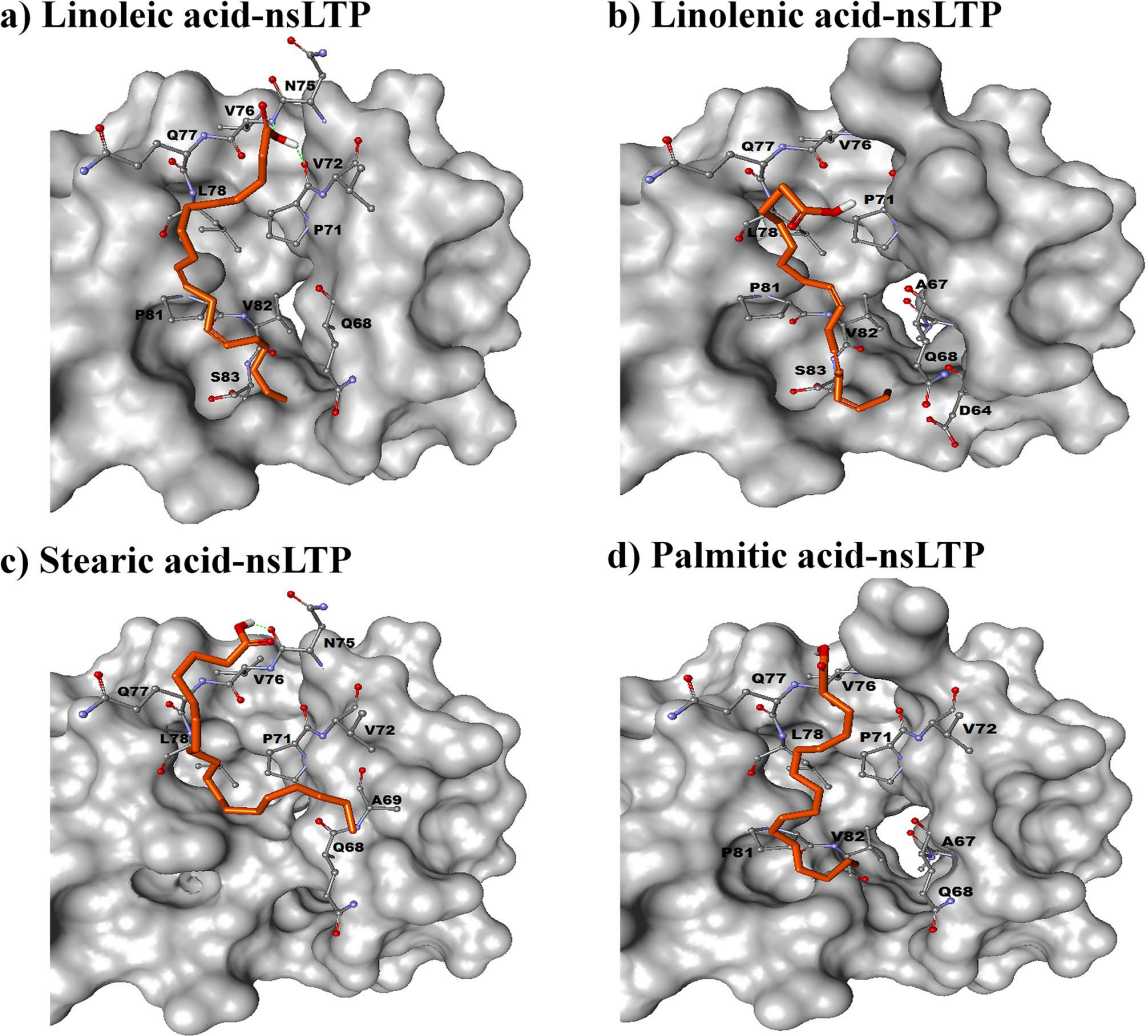
Molecular docking of lipid molecules on nsLTP1 from fennel (*Foeniculum vulgare*) seeds). Best docked conformations of fennel nsLTP1 with Linoleic acid (**a**), Linolenic acid (**b**), Stearic acid **(c**), and Palmitic acid (**d**) as produced by AutoDock Vina. Interacting residues are labeled and shown in ball and stick conformation. The poses were generated through Discovery Studio Visualizer.

The best-docked conformation of each of the lipids with fennel nsLTP1 is presented as Fig. 6, which depicts the docked conformation of linoleic acid, linolenic acid, stearic, and palmitic acid in the binding pocket of fennel nsLTP1. The poses of lipid molecules were mainly considered within the active site of nsLTP1, as well as their binding scores for ranking the lipids. Moreover, Supplementary Table S2 represents the output of AutoDock Vina in terms of binding affinity (Kcal/mol) and interactions found between protein and ligands.

### In vitro lipid binding activity of fennel nsLTP1

**Figure 7:**
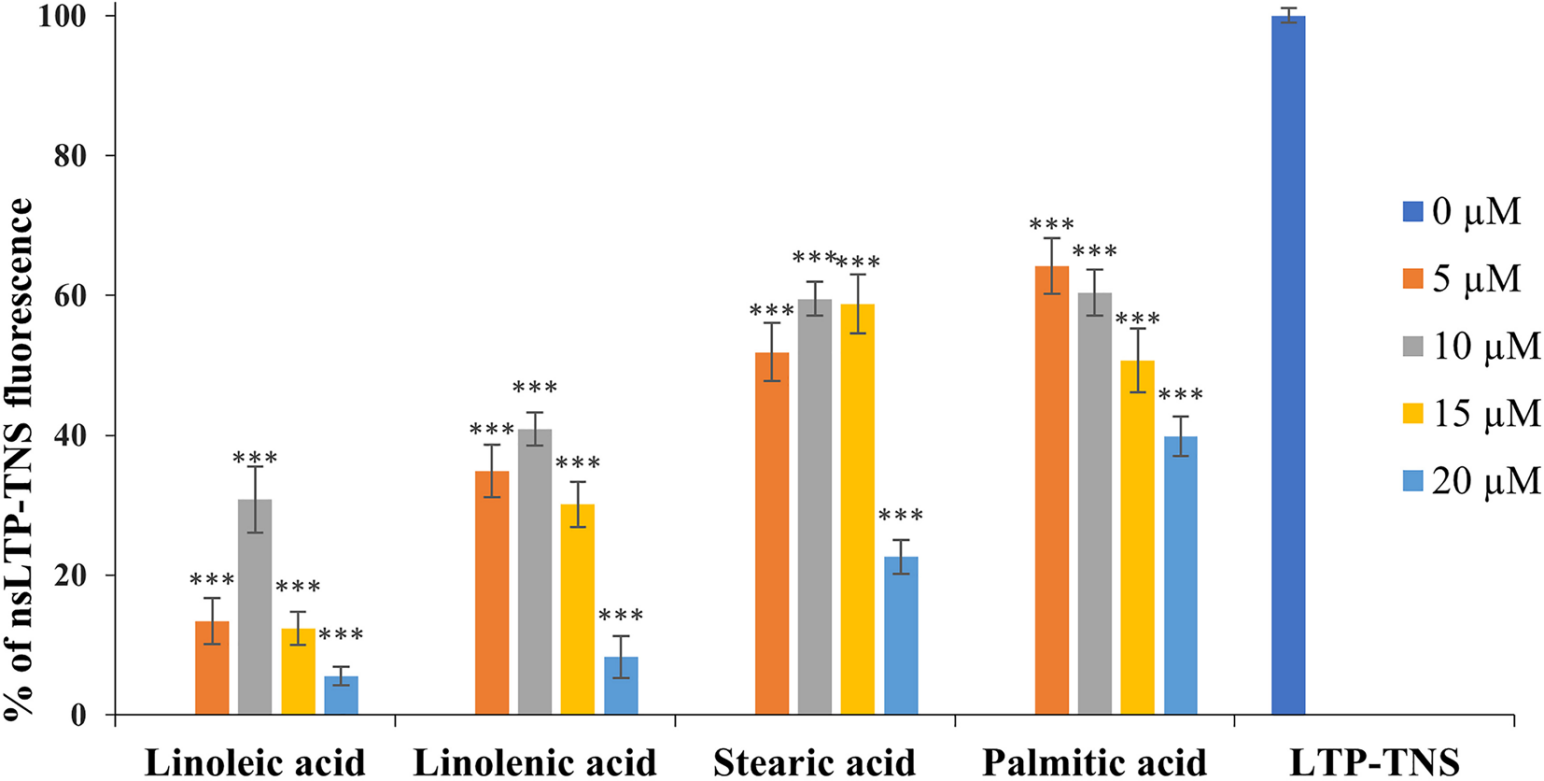
The effect of fatty acids on the fluorescence level of fennel nsLTP1-TNS complex. The binding ability of FAs to compete with TNS (3.5 µM) for binding to fennel nsLTP1 (4 µM) at varying concentrations (5 µM, 10 µM, 15 µM, and 20 µM). The ability of the FAs to compete with TNS for binding to fennel nsLTP1 was shown by the reduction of fluorescence from the nsLTP1-TNS complex. Data from three independent experiments are presented with mean and standard deviation (S.D.) highlighted as *P < 0.05, **P < 0.01, ***P < 0.001.

To evaluate the lipid-binding ability of fennel nsLTP1, the fluorescence-based assay was performed using 6-p-Toluidino-2-naphthalenesulfonic acid (TNS), a lipophilic probe that fluoresces as a result of binding in a hydrophobic environment^35,36^. Lipids used in the study include saturated fatty acids (FAs) such as stearic acid and palmitic acid with the chain length of C18 and C16 respectively; as well as polyunsaturated FAs involving linoleic acid (cis, cis-9,12-Octadecadienoic acid) and linolenic acid (all-cis-9,12,15-octadecatrienoic acid) with the chain length of C18. The binding of TNS to the hydrophobic cavity of nsLTP1 accounted for the highest fluorescent intensity, whereas the addition of nsLTP1 to the mixture of TNS and FAs decreased its fluorescence (Fig. 7).

Interestingly, among the FAs in the experiment, linoleic acid displaced the TNS probe with the highest efficiency (4–40% of the control fluorescence) at all the concentrations tested. Linolenic acid, on the other hand, exhibited lesser efficiency (7–59% of the control fluorescence) as compared to linoleic acid in displacing the TNS probe from the hydrophobic cavity of nsLTP1. In contrast, stearic acid showed lower displacement efficiency (21–59% of the control fluorescence) than linolenic acid but slightly higher efficiency than palmitic acid (40–69% of the control fluorescence). Besides, palmitic acid was shown to reduce the fluorescent intensity in a dose-dependent manner. However, the other FAs caused an initial increase in intensity, followed by a gradual decrease as the FA concentration was increased.

### Cytotoxic effect of fennel ns-LTP on human breast cancer cell line

The screening of fennel nsLTP1, as depicted in (Supplementary Figure S5a) suppressed MCF-7 human breast cancer cells in a dose-dependent manner compared to untreated control cells with the IC_50_ value of 6.98 µM. At the lowest concentration tested (5 µM), fennel nsLTP1 reduced statistically significant (***P < 0.001) number of viable MCF-7 cells with respect to untreated cells with percentage inhibition of 31.8%. Whereas, at highest concentration (150 µM), MCF-7 proliferation was inhibited significantly (***P < 0.001) by 77.1%. Moreover, doxorubicin (positive control) resulted in a dose-dependent inhibition with an IC_50_ value of 0.037 µM (Supplementary Figure S5b).

Fennel nsLTP1 treated MCF-7 cells were further examined for morphological alteration after 48 h using phase-contrast microscopy. While the untreated cells were observed to have a monolayer with a high density, the doxorubicin and nsLTP1 treated cells, however, displayed morphological alterations and reduced cell count (Supplementary Figure S5c). The number of viable cells decreased as the concentration of the fennel nsLTP increased. Moreover, the nsLTP1 treated cells demonstrated roundedness and shrinkage, suggesting the antiproliferative effect of the protein. It was compared with doxorubicin treated cells where a significantly low number of cell count was noted in addition to morphological alteration.

## Discussion

Since their discovery thirty years ago, structural studies of nsLTP1 from various plants have drawn several research interests to understand their biological role. In higher plants, the nsLTPs are that account for 4% of the total soluble proteins^1^. They are cationic, cysteine-rich, small molecular weight proteins with sizes ranging from 6 to 10 kDa^1,8^. The conserved eight cysteine residues that form four disulfide bridges stabilize the three-dimensional structure, as well as the presence of an internal hydrophobic cavity, which is regarded as a distinctive structural feature of nsLTP1s^1,6,8^. In the present study, therefore, nsLTP1 from fennel seeds was isolated and characterized for the first time; following another research that identified it as a small molecular weight protein claimed to be responsible for allergenicity of fennel in some people^37^. Fennel nsLTP1 is the sixth nsLTP1 reported from the Umbelliferae family after carrot, celery, cumin, dill, and ajwain.

Due to the fact that plant nsLTP1s have basic character (isoelectric point around 9) and relatively small molecular weight, the use of salts that reduce their solubility offers the advantage of successful precipitation devoid of denaturing their native conformation^38^. Thus, precipitation of fennel seed proteins using 60% ammonium sulfate is found to be a well-suited protocol for efficient isolation and purification of nsLTP1 using size exclusion chromatography (Fig. 1a), RP-HPLC (Fig. 1b), and molecular weight confirmation by MALDI-TOF mass spectrometry (Fig. 1c). Unlike nsLTP1s from tomato^39^ and human serum^40^, which were difficult to isolate the pure form due to their tight association with other proteins, nsLTP1 from fennel was identified as unbound form, not associated with other proteins.

Fennel nsLTP1 is a monomeric protein chain of 91 amino acid residues, with a total of 8 conserved cysteines located at the positions 4, 14, 28, 29, 49, 51, 74, and 88 that are engaged in the formation of disulfide bridges enabling the same 3D structure known for a number of nsLTPs. Previous studies show that the disulfide bond linkages take place between Cys^I^–Cys^VI^, Cys^II^–Cys^III^, Cys^IV^–Cys^VII^, and Cys^V^–Cys^VIII^, where the –Cys^V^-X-Cys^VI^–motif usually have a hydrophilic residue like asparagine in the case of fennel nsLTP1^41,42^. The conserved hydrophilic residues are essential for the biological function of the protein in plants^43^.

Several conserved hydrophobic amino acid residues involving Val7, Leu11, Pro13, Val15, Leu18, Val32, Ile62, Ala67, Leu70, Pro71, Val76, Leu78, Ile80, Pro81, Val82, Ile83, and Pro84 are observed in fennel nsLTP1. According to structural studies of nsLTP1s from other plants, these hydrophobic residues are buried within the molecule, not interacting with each other creating a suitable environment for forming a large internal tunnel-like cavity that provides a potential binding site for hydrophobic ligands^18,44^. The presence of conserved residues, including proline and glycine, are also noted, which, according to previous studies, contribute to the conservation of a particular protein fold^25^. Moreover, aromatic amino acid residues, including tryptophan and phenylalanine, are absent in fennel nsLTP1; several plant nsLTP1s are also reported to lack tryptophan^7^. There are two tyrosine residues at the positions of Tyr17 and Tyr59 that are conserved in many plant nsLTP1s as well^10^. In Solanum *molenga,* however, the Tyr17 is replaced by Phenylalanine. Thus, the complete amino acid sequence of fennel nsLTP1 depicted the overall molecular composition of the protein; consequently, it led the research interest to further analyze its 3D structure for a better understanding of its biological function.

Owing to the fact that the function of protein is determined by its structure, the 3D structure of proteins is crucial in terms of defining the biological pathways^45^. The computational method is a commonly used approach for predicting the structure of proteins first by identifying a homologous relationship with a known structure to generate the model structure^46^. Therefore, the 3D-structural modeling of fennel nsLTP1 is established using the crystal structure coordinates of nsLTP1 from eggplant (*S*. *melongena*) 5TVI as a template. 5TVI is selected as the best template based on BLASTp result; it is 59.34% identical and 74% similar to fennel nsLTP1 with 100% query coverage and e-value of 3E−33. The homology modeling, therefore, indicated that the structure of fennel nsLTP1 has four α-helices: H1 (4–20), H2 (26–38), H3 (42–57) and H4 (64–74); a long C-terminal tail (75–91); and three short loops at the positions of (21–25), (37–41), (58–63) residues. The study from the crystal structure of 5TVI depicted that these helices are engaged in the form of a hydrophobic cavity, where the overall structure is stabilized by the presence of four disulfide bonds that exist between cysteine (4–51), (14–28), (29–74) and (49–88) that are also retained by fennel nsLTP1 structure^16^. The internal hydrophobic cavity is regarded as a possible site for the binding of lipid^16^. Studies from other plant nsLTP1s indicated that the long C-terminal tail is crucial for the orientation of the lipids in the cavity^16,47^.

Despite the presence of nsLTP1s with similar spatial structures from diverse plant species, they exhibit distinct ligand-binding behaviors^48,49^. It is reported that nsLTP1s from different plants show variable binding affinities to the tested FAs^36,48,50^. Accordingly, the observed binding preferences of nsLTP1s to bind FAs are not characteristic of every plant nsLTP1s^51,52^. For instance, no FA binding was observed in antimicrobial protein Ace-AMP1 from onion; selective binding of saturated FAs to Xylem Sap Protein (XSP10) from tomato was noted, and dill (*Anethum graveolens)* lipid transfer protein (AG-LTP) exhibited a lack of selectivity toward various FAs. These characteristics could signify the variety of roles LTP play in plants, including defense against biotic stress, transportation of lipids, as well as cutin and suberin wax protective layers formation^49,53^.

In the present in vitro lipid binding study using TNS fluorescent probe, nsLTP1 from fennel binds to both saturated and unsaturated FAs with variable efficiency. Similar variability in the binding affinities is observed in the study of nsLTP1s from ginkgo (*Ginkgo biloba*), lentil (*Lens culinaris*) seeds, *Nicotiana tabacum,* and dill (*Anethum graveolens*); where nsLTP1s were observed to have more preference to unsaturated FAs^36,48,49,53^. This can be explained in terms of FA chain length, the number as well as the position of double bonds, that could determine the way FAs are accommodated in the hydrophobic cavity of the protein^49,54,55^. It appeared that palmitic acid was the least efficient in displacing TNS from the binding pocket of fennel nsLTP1 as compared to stearic acid at all tested concentrations (Fig. 7). As supported by previous lipid binding studies, saturated FAs with relatively shorter chain length allow a limited number of hydrophobic interactions resulting in relatively lower binding efficiency as compared to saturated FAs with longer chain length^47,56,57^.

On the other hand, significantly higher binding efficiency (***P < 0.001) was noted for linoleic acid, followed by linolenic acid (Fig. 7). The presence of one cis-double bond in FAs with a chain length of C18 (as in linoleic acid) has a higher affinity for the protein as it enforces the FA to a curved shape for easier accommodation in the binding site^23,56–58^. Moreover, the binding assay revealed an initial increase in the percentage of fennel nsLTP1-TNS fluorescence at the lower concentration of displacing FAs, followed by a decrease in the intensity at higher FA concentration (Fig. 7). This is in line with Gb-nsLTP1 from *Ginkgo biloba*, Jug r 3 from walnut and wheat LTP, where a similar biphasic change in the intensity of fluorescence in response to increased concentration of FAs was observed^36,50,58^. Such a change could be explained in terms of the binding of FA in the cavity along with the fluorescent probe that increases the fluorescence intensity as a result of effective solvent exclusion from the binding pocket.

Further increase in the FA then displaces the probe leading to an observed decrease in intensity^23,50^. The binding affinity of fennel nsLTP1 to the FAs is also an important factor that determines how readily TNS is displaced from the hydrophobic cavity as the FA concentration increases^50^. Although the lipid binding assay is performed on a fewer number of FAs as a preliminary study which lacks kinetic and mechanistic analysis, we think that the outcome is still in line with the proposed role of nsLTP1 to bind lipids.

Molecular docking was employed to examine further the lipid-binding behavior of fennel nsLTP1 using its predicted 3D structure. The same FAs used in the wet lab experiment were selected as ligands. Protein–ligand interactions were observed by Discovery Studio Visualizer. The binding pocket is identified to be located at the residues between 61 and 91; where the lipid molecules are surrounded by helix H4 and C-terminal region. A similar result has been obtained in a lipid-binding study employing the crystal structure of rice nsLTP1 where H4 and the C‐terminal loop region is demonstrated as the binding pocket with the most hydrophobic interactions spotted^59^. Moreover, the aliphatic parts of the FAs are observed to be situated in the hydrophobic binding pocket of fennel nsLTP1, while their hydrophilic heads exhibited distinct orientations. Besides, the residues involved in lipid interactions include Asp64, Ala67, Gln68, Ala69, Pro71, Val72, Asn75, Val76, Gln77, Leu78, Pro81, Val82, and Ser83; where the common interacting residues observed among all studied molecules were Gln68, Pro71, Gln77, and Leu78. Therefore, it is suggested that the major lipid binding forces are hydrophobic interactions that are stabilized through hydrogen bonds between the protein and polar head of the lipid^10,17,59^.

Moreover, the output of AutoDock Vina (Supplementary Table S2) indicated that the order of binding affinity among the four studied FAs is observed as linoleic acid > linolenic acid > Stearic acid > Palmitic acid, which agreed with wet-lab results. The same order of binding affinity with four lipid molecules was reported in the nsLTP1 of *Lotus japonicus*^60^. Earlier experimental studies also revealed that linoleic acid exhibited greater binding affinity than linolenic acid^61^. The lipid-binding property of plant nsLTP1s is ascribed to the two highly conserved consensus sequences, T/SXXDR/K and PYXIS^62^. In fennel nsLTP1, the first pattern of the motif is TPQDK with the residues from 41 to 45, whereas the second motif PVSRT residues from 81 to 84. Among the two consensus sequences, Pro81, Val82, and Ser83 within the second conserved motif at the C-terminal loop region is found to be involved in lipid binding. Our docking studies, therefore, suggested that linoleic acid exhibited the highest binding affinity among all molecules studied, confirming results obtained through wet-lab experiments.

Plant nsLTPs have capability to bind a variety of lipids such as FAs, fatty acyl CoA, phospholipids, glycolipids, etc^1^. This variation makes them capable of performing different functions^63^. It has also been suggested that nsLTPs may have multiple binding sites (lipid binding interactions)^17^. Structural studies have revealed that the protein may have elasticity in the presence of ligand^64^. In the current study, polar heads of all studied FAs were directed toward helix 4, making hydrophobic interactions with residues in the vicinity. Closer examination of ligand protein interaction, however, showed variation among FAs. Linoleic acid is found to be involved in making hydrogen bond with two residues P71 and V76, whereas stearic acid is making one hydrogen bond with N75 of fennel. All fatty acids are making alkyl interactions with fennel nsLTP1 (Supplementary table S3). It has already been documented that nsLTPs have shown more affinity for unsaturated FAs as compared to saturated^36,48,49,53^ and our results are in line with these observations.

Earlier literature indicates studies on nsLTP1 from different fruits and nuts. We have aligned fennel nsLTP1 sequence with peach, hazelnut, and walnut sequences and observed 37.78%, 34.07%, and 35.16% sequence similarity, respectively (Supplementary Figure S4). Intramolecular interaction of peach and walnut nsLTP1 in the presence and absence of oleic acid has been studied extensively. Critical residues in the binding pocket of peach nsLTP1 that are affected due to Oleic acid interaction range for 76 to 86^64^. A similar binding pocket is retained in the case of fennel nsLTP1 as well. Among the four common interacting residues observed in our study (Gln68, Pro71, Gln77, Leu78), only Pro71 interaction was found to be retained in peach nsLTP1-Oleic acid complex^64^. Pro71 is conserved in most of the nsLTP1 sequences^65^ and is likely to be involved in ligand interaction. Another residue contributing in ligand–protein interaction is L78 in Fennel nsLTP1, which is consistent with the earlier observation in peach and walnut where the ligand–protein interaction at this position is retained, albeit L is replaced by I and V in peach and walnut, respectively^58^.

An interesting aspect of nsLTPs is their capability to act as allergens, which have been studied extensively in peach^37^ and walnut^58^. Four IgE binding epitope regions in peach nsLTP1 (17–25, 41–48, 65–69, 77–91) have been identified, which, if retained, are likely to elicit a strong allergic response^66^. Apple nsLTP1 retained epitope composition and showed higher IgE binding affinity in comparison to hazelnut and sunflower nsLTP1 sequences, which showed more variation and lower IgE binding affinities ^65^. As far as fennel is concerned, studies of allergens from fennel are limited to date. The analysis of fennel allergy among peach sensitive patients concluded that patients with peach allergy are likely to develop severe hypersensitivity to fennel^37^. To further explore the allergenic potential of fennel, we compared the fennel and peach nsLTP1 sequences and observed that IgE binding epitope regions are not highly conserved in fennel; hence attributing allergic response solely to fennel nsLTP1 would be unfair. It is noteworthy that apart from nsLTP1, another protein, Pathogenesis related protein 1 (PRP1), has been identified as a possible allergen for fennel sensitivity among peach allergic patients^37^. Nevertheless, the possible role of fennel nsLTP1 as an allergen needs to be elucidated further.

The study further demonstrates the ability of fennel nsLTP1 to inhibit the proliferation of MCF-7 breast cancer cell lines for the first time (Supplementary Figure S5a), and the result was compared with the known potent anticancer drug, doxorubicin (Supplementary Figure S5b). When exposed to fennel nsLTP1, MCF-7 cells lost their proliferative ability in a dose-dependent manner. Moreover, the antiproliferative effect of fennel nsLTP1 was evident from the reduced number of viable cells, which explains its capability to induce cellular death. Compared to the calculated fennel nsLTP1 IC_50_ (6.98 µM), LTP isolated from *Brassica campestris* exhibited antiproliferative activity on MCF-7 breast cancer cell lines, the IC_50_ value of 1.6 µM. Besides, the same LTP from *Brassica campestris* showed inhibitory activity against hepatoma Hep G2 cells with an IC_50_ value of 5.8 µM^67^, indicating that the antiproliferative activity of LTPs from the same plant varies based on the type of cell line tested. In contrast, nsLTP from Mung bean was shown to be devoid of an antiproliferative effect on MCF-7 cancer cell lines. This variability in antiproliferative behavior was proposed due to the difference in the amino acid sequence and the specific portion of the same protein responsible for the observed activity^67^.

A closer examination of fennel nsLTP1 treated cells by phase-contrast microscopy further confirmed a dose-dependent decrease in a cell count as well as consequent morphological changes (Supplementary Figure S5c). These include cellular roundness and shrinkage that is regarded as a morphological alteration related to apoptosis^68,69^. Our studies progress to apoptosis analysis using flow cytometry to better understand the mechanism of fennel nsLTP1 to kill MCF-7 breast cancer cell lines. The subsequent gene expression analysis evidenced that apoptosis is the possible cell killing mechanism.

## Conclusion

The present study determined the isolation, purification, and amino acid sequence of nsLTP1 from fennel (*Foeniculum vulgare*) seeds. Its 3D structure established in silico using the crystal structure coordinates of nsLTP1 from eggplant (*S*. *melongena*) 5TVI as a template. These structural analyses depicted that fennel nsLTP1 contains eight cysteine residues responsible for the disulfide bridges and a hydrophobic cavity. Moreover, in vitro lipid binding efficiency revealed that fennel nsLTP1 has the lipid-binding ability. A molecular docking study was also performed to validate the wet lab in vitro lipid binding study using its predicted 3D model structure. It is of future research interest to study the kinetics of lipid binding with a wide range of FAs as well as the mode of action. Additionally, this study reports a preliminary antiproliferative activity of fennel nsLTP1 against MCF-7 human breast cancer cell lines for the first time.

## Materials and methods

### Extraction of proteins

Fennel (*Foeniculum vulgare*) seeds were ground, and the powder was defatted using *n*-hexane for 24 h. Then the hexane was filtered out and was dried in the hood. The dry powder was then soaked in 20 mM Tris/HCl*, p*H 8 under continuous stirring for four days at 4 °C, and filtered. The filtrate obtained was centrifuged at 40,000 rpm for 30 min, followed by precipitation of the supernatant using 60% ammonium sulfate at 4 °C. Finally, the precipitated crude protein extract was centrifuged at 14,000 rpm. The pellet was dissolved and dialyzed in water for two days at 4 °C and lyophilized.

### Gel electrophoresis

Polyacrylamide gel electrophoresis (Tris-tricine 12% separation and 4% stacking gel were utilized throughout the study for the analysis of crude and/ isolated protein at constant 200 V for 1 h. The gels were stained using Coomassie stain followed by destaining water ^70^.

### Gel filtration chromatography

HiPrep Sephacryl S-200 HR16/20 column was used as a first-dimensional chromatography to separate the crude protein extract using AKTA start FPLC. The column was first equilibrated with 20 mM Tris/HCl, pH 8. The sample elution was performed with the same buffer at the flow rate of 5 ml/min, and absorbance monitored at 280 nm.

### Reverse-phase chromatography

The RP-HPLC was utilized to purify further the pooled fractions obtained from gel filtration chromatography using Aeris Widepore 3.6UXB-C18 (150 × 2.1 mm) column. The column was equilibrated with 0.1% TFA-water (solvent A). The elution of protein was performed at the flow rate of 1 ml/min, using a gradient of acetonitrile from 0 to 60% in 60 min, where the absorbance was measured at 214 nm.

### MALDI-TOF mass spectrometry

The purified intact nsLTP1 was analyzed by MALDI-TOF mass spectrometer (Autoflex Speed, Bruker, USA). A 1 µl of the matrix 3,5-Dimethoxy-4-hydroxycinnamic acid (SPA) prepared in 50% acetonitrile–water containing 0.1%TFA, and 1 µl of the sample in 0.1% TFA-water were mixed and spotted on the MALDI plate. The Flex-control software (Bruker, USA) was used for the analysis of the spectra.

### Protein modification by vinyl pyridine

The purified and lyophilized protein (10 mg) was mixed with 10 µl of reduction and alkylation buffer (Guanidine/HCl 6 M, tris base 0.2 M, di-sodium EDTA, 2 mM, and 5 μl of 2-Mercaptoethanol) in a ratio of 1:1. Then, 2-mercaptoethanol in 1:10 ratio was added to the solution under nitrogen and incubated at 50 °C for 4 h. The 4-vinyl pyridine was then added to the solution in a 1:1 ratio and incubated for 1.5 h at 37 °C. The reaction was finally quenched with 2-mercaptoethanol and 5% acetic acid in a 1:1 ratio with the same volume as R&A buffer added to dissolve the protein. The modified protein was dialyzed against deionized water for 24 h and vacuum dried. Finally, the modified lyophilized sample was dissolved in Tris/HCl 0.1 M *pH* 8.5 and purified using RP-HPLC^71^.

### Trypsin digestion and peptide fingerprinting

The modified fennel nsLTP1 was digested with TPCK (Tosyl-phenylalanyl-chloromethyl-ketone) treated trypsin (20:1 ratio) in 50 mM Tris/HCl pH 8 for 4 h at room temperature^72^. Then, the solution was titrated with 1 M acetic acid to pH 4, and the resulting digested protein fragments were separated by RP-HPLC using column (Aeris Widepore 3.6UXB-C18 150 × 2.1 mm) with gradient elution of 0–60% B (0.1% TFA-acetonitrile) in 55 min. The flow rate was set at 1 ml/min, and absorbance monitored at 214 nm.

### Cleavage with CNBr

The methionyl bond of purified intact fennel nsLTP1 was cleaved with CNBr^73^. The protein (1 mg) was dissolved in 2 ml of 70% formic acid. Then 4 mg of CNBr was added to the solution at 25 °C and incubated in the dark for 24 h. The resulting protein digest was SpeedVac dried for further purification by RP-HPLC using Aeris Widepore 3.6UXB-C18 150 × 2.1 mm column with gradient elution 0–60% B (0.1% TFA-acetonitrile) in 55 min. The flow rate was set at 1 ml/min, and absorbance at 214 nm.

### Amino acid sequencing

The *N*-terminal amino acid sequence of the intact protein, and peptides, was analyzed using protein sequencer^74^, with an online Phenylthiohydantoin (PTH) analyzer model PPSQ33A (Shimadzu).

### Lipid binding study

The lipid-binding ability of fennel nsLTP1 was evaluated using TNS, a fluorescent probe with a slightly modified protocol^53^. The fluorescence experiment was performed using the Ultramax M5 microplate reader (Molecular Devices) at 25 °C. The intensity of fluorescence was measured at an emission wavelength of 437 nm and excitation of 320 nm. Prior to the initial fluorescence record (**F**_**o**_), 3.5 µM TNS was incubated with and without lipids for 1 min in 1 ml buffer containing 175 mM mannitol, 0.5 mM K_2_SO_4_, 0.5 mM CaCl_2_, 5 mM MES, pH 7.0. All the FAs were prepared in Dimethyl sulfoxide (DMSO) at varied concentrations of 5 µM, 10 µM, 15 µM, and 20 µM. Then, nsLTP1 (4 µM) was added, and fluorescence was recorded after 2 min of incubation (F). The percentage of nsLTP-TNS complex fluorescence was calculated as:

1 $$\frac{(\mathrm{F}-\mathrm{Fo})}{\mathrm{Fc}}\times 100$$

where F_c_ is the fluorescence of the nsLTP-TNS complex in the absence of lipid.

### Structural modeling

The three-dimensional structure of fennel nsLTP1 was predicted using a homology modeling approach. The amino acid sequence identified in the current study was used to search for a template against Protein Data Bank (PDB) using an online protein BLAST program (https://blast.ncbi.nlm.nih.gov/Blast.cgi). The best match was found to be with nsLTP1 from *Solanum melongena* (PDB ID: 5TVI), having 59.34% identity with the target sequence; hence it was selected as a template. Multiple sequence alignment was performed through Clustal Omega^75^. Crystal structure coordinates of 5TVI were used to construct the three-dimensional model of fennel nsLTP1 using Modeller version 9.23^76^. The modeled structure was evaluated using PROCHECK and PROSA^77,78^. To examine the structural variation between target and template, the modeled structure of fennel was superimposed with the crystal structure of 5TVI using UCSF Chimera version 1.14^79^.

### Protein and ligand preparation

To examine the interaction between nsLTP1 and different FAs, a modeled three-dimensional structure of fennel nsLTP1 was docked with linolenic, stearic, palmitic, and linoleic acids. Before docking studies, protein and ligand files were prepared. Ligand files of palmitic acid (CID: 985), linoleic acid (CID: 5280450), and linolenic acid (CID: 5280934) were downloaded from PubChem Database^80^. The structure of stearic acid (ID: 5091) was downloaded from ChemSpider^81^. All these structures were converted into PDB format through OpenBabel command-line interface to be utilized by docking software^82^. The protein structure was prepared by adding polar hydrogens through AutoDockTools (ADT) and was saved in Protein Data Bank, partial charge (Q), and Atom Type (T) (PDBQT) format.

### Molecular docking

The four lipid molecules were docked on protein structure using AutoDock Vina program^83^. The binding pocket of the protein was identified using the Prosite tool^84^. The grid box was set around the binding pocket of protein with grid points 80 Å × 46 Å × 80 Å. Different possible conformations with binding energies were obtained with each round of docking, and the best binding pose in all docked conformations was chosen for each ligand. Discovery Studio Visualizer was used to visualize the AutoDock Vina output^85^.

### Cell culture

The cytotoxic activity of fennel nsLTP1 was examined on MCF-7 (ATCC HTB-22) human breast cancer cell line that was purchased from the American Type Culture Collection (Manassas, VA). Dulbecco's Modified Eagle Medium (DMEM) was purchased from Life Technologies Corporation (ThermoFisher Scientific, Carlsbad, CA). The cultured cells were grown as a monolayer in DMEM that is supplemented with 10% (v/v) fetal bovine serum (FBS) and 1% (v/v) penicillin–streptomycin at 37 °C in an incubator at 95% humidity and 5% CO2.

### Cell proliferation assay

In vitro antiproliferative effect of fennel nsLTP1 against MCF-7 breast cancer cell lines was examined using MTT (3-(4,5-dimethylthiazol-2-yl)-2,5-diphenyltetrazolium bromide or MTT) assay that measures the metabolic activity of cells as a marker for cellular viability^86^. MCF-7 cells seeded at the density of 10,000 cells/well incubated in 96-well plate at 37 °C in 5% CO_2_ in the absence or presence of the samples for 48 h at the final volume of 200 µl. The medium was then removed post-treatment for the indicated time, and MTT dye (0.5 mg/ml in PBS) was added to the final volume of 200 µl. Following incubation at 37 °C in 5% CO_2_ for 4 h, the dye was discarded, and the purple-blue colored formazan crystal was solubilized in DMSO (100 µl). The plate was read for optical density (OD) using a UV spectrophotometer (SpectraMax M5 Microplate Reader) at the wavelength of 570 nm to determine the mitochondrial activity. The study was performed in three independent experiments with triplicates. The percentage of inhibition was calculated using the formula:

$$\frac{\mathrm{Absorbance of untreated cells}-\mathrm{Absorbance of treated cell}}{\mathrm{Absorbance of untreated cells}}*100$$

### IC_50_ determination

The dose–response curve was used to extrapolate the IC_50_ value from the three independent triplicate experiments over the range of tested concentrations by nonlinear regression analysis using Prism version 8.0.0 for Windows, GraphPad Software, San Diego, California USA.

### Statistical analysis

The experimental data were analyzed using the R statistical programming language, version 3.5.1^87^. Primarily, the statistical requirements of continuous variables for parametric statistical tests were determined. One-way ANOVA (analysis of variance) followed by Tukey’s post-hoc test was used to assess the statistical significance for comparing two groups or multiple groups. Results with *P* values of < 0.05 were considered statistically significant. The significance was presented as *P < 0.05, **P < 0.01, ***P < 0.001.

## Supplementary information

Supplementary Information.
