# Supplementary material for: Structural characterization and in vitro lipid binding studies of non-specific lipid transfer protein 1 (nsLTP1) from fennel (Foeniculum vulgare) seeds
Source: Sci Rep. 2020 Dec 4;10:21243. doi: 10.1038/s41598-020-77278-6 (PMC7718255; doi:10.1038/s41598-020-77278-6)
Supplement: Supplementary file 1 — Supplementary Information. [file 41598_2020_77278_MOESM1_ESM.pdf]

Supplementary information

Structural Characterization and *in vitro* Lipid Binding Studies of Nonspecific Lipid Transfer Protein 1 (nsLTP1) from Fennel (*Foeniculum vulgare*) Seeds

Mekdes Megeressa[1], Bushra Siraj[2], Shamshad Zarina[2], Aftab Ahmed[1*]

1. Biomedical and Pharmaceutical Sciences, Chapman University School of Pharmacy, Irvine, CA 92618, USA
2. Dr. Zafar H. Zaidi Center for Proteomics, University of Karachi, Karachi 75270, Pakistan

*Corresponding author

Aftab Ahmed
Chapman University School of Pharmacy
9401 Jeronimo Road
Irvine, CA 92618
USA
Tel: (714) 516-5465
Fax: (714) 516-5481
aahmed@chapman.edu

Supplementary Table S1: Top-ranked template sequences obtained by BLASTp results against PDB database.

S. No.	PDB ID	Organism Name	Total score	Query coverage	Max. Identity	Positives	E-value
1.	5TVI_V	*Solanum melongena*	119	100%	59.34	74.73	9.54E-37
2.	2MAL_A	*Lens culinaris*	78.6	100%	43.95	59.34	2.46E-20
3.	5LQV_A	*Lens culinaris*	77	98%	43.33	58.89	9.44E-20
4.	1BWO_A	*Triticum aestivum*	74.7	98%	37.77	58.89	5.52E-19
5.	2ALG_A	*Prunus persica*	73.2	98%	37.77	58.89	2.46E-18
6.	1CZ2_A	*Triticum aestivum*	73.2	97%	37.07	58.43	2.69E-18
7.	1BV2_A	*Oryza sativa*	70.1	98%	37.77	56.67	5.14E-17
8.	1AFH_A	*Zea mays*	67	100%	38.04	56.52	6.78E-16
9.	2N81_A	*Pisum sativum*	67	100%	40.86	56.99	8.76E-16
10.	1BE2_A	*Hordeum vulgare*	65.5	98%	33.33	56.67	2.96E-15
11.	1SIY_A	*Vigna radiata var. radiate*	63.9	98%	36.667	55.56	1.04E-14
12.	4XUW_A	*Corylus avellana*	63.5	100%	34.066	52.75	1.46E-14
13.	2N2Z_A	*Anethum graveolens*	61.6	98%	36.957	54.35	8.98E-14
14.	6FRR_A	*Artemisia vulgaris*	61.2	98%	33.333	50	1.35E-13
15.	1T12_A	*Nicotiana tabacum*	58.9	100%	36.264	52.75	1.02E-12

Supplementary Table S2: Molecular docking results of lipid molecules on nsLTP from fennel (*Foeniculum Vulgare*) seeds using AutoDock vina

Compounds	Autodock Vina score	Ligand binding site interacting residues	
		Common Residues	Unique residues
Linoleic acid	-3.2 Kcal/mol	Q68, P71, Q77, L78	V72, N75, V76, Q77, P81, V82, S83
Linolenic acid	-2.9 Kcal/mol	Q68, P71, Q77, L78,	D64, A67, P81, V82, S83
Stearic acid	-2.4 Kcal/mol	Q68, P71, Q77, L78,	A69, V72, N75, V76
Palmitic acid	-2.4 Kcal/mol	Q68, P71, Q77, L78,	A67, V72, V76, P81, V82

Supplementary Table S3: Contribution of non-covalent interactions (Hydrogen bond, hydrophobic interaction) in protein-fatty acid complex.

Compounds	Hydrogen bond (Å)	Hydrophobic interactions
Linoleic acid	P71:N – lig:O (2.3 Å)<br>V76:N – lig:O (3.0 Å)	P71 (Alkyl) (4.8 Å)<br>P81 (Alkyl) (4.1 Å)
Linolenic acid	-	A67 (Alkyl) (3.9 Å)<br>P71 (Alkyl) (5.4 Å)<br>L78 (Alkyl) (5.1 Å)<br>P81 (Alkyl) (5.0 Å)
Stearic acid	N75:O – lig: H (2.2 Å)	P71 (Alkyl) (5.2 Å)<br>V72 (Alkyl) (4.8 Å)
Palmitic acid	-	P71 (Alkyl) (5.1 Å)<br>P81 (Alkyl) (5.3 Å)

Supplementary Figure S1: Electrophoretic profile of gel filtration fractions of fennel seed proteins by 12 % Tris/Tricine gel.Lane 1: Molecular weight markers. Lane 2: Crude protein. Lane 3-11: pooled gel filtration fractions 21-23, 24-26, 27-29, 30-32, 33-35, 36-38, 39-41, 42-43, and 44-45. The arrow indicates a band for nsLTP.

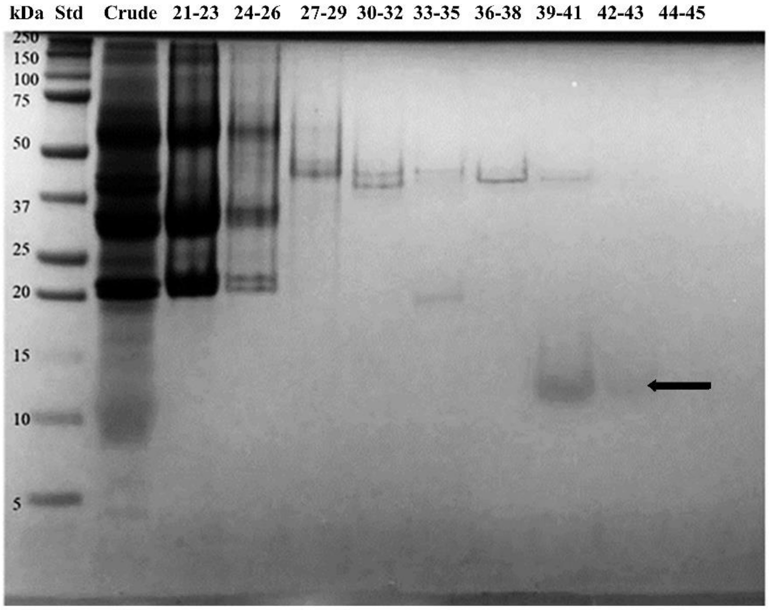

Supplementary Figure S2: Top-ranked template sequences for multiple sequence alignment obtained through BLASTp search against PDB database.

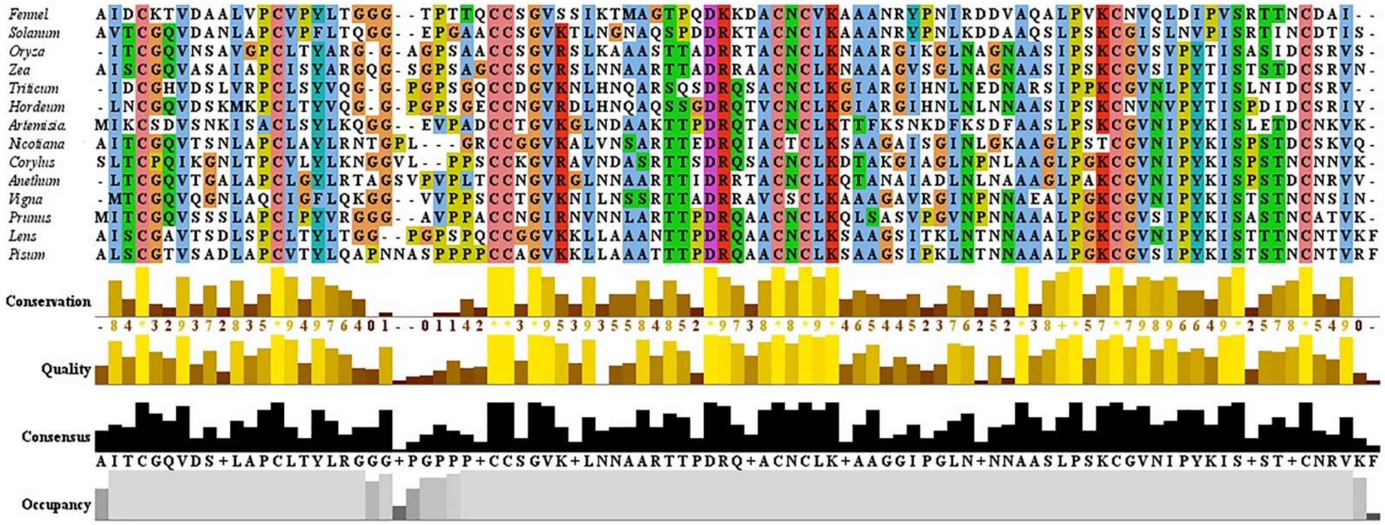

Supplementary Figure S3: Ramachandran Plot of modeled nsLTP1 from fennel seeds. More than 90% of residues appear in allowed regions suggesting a good quality model.

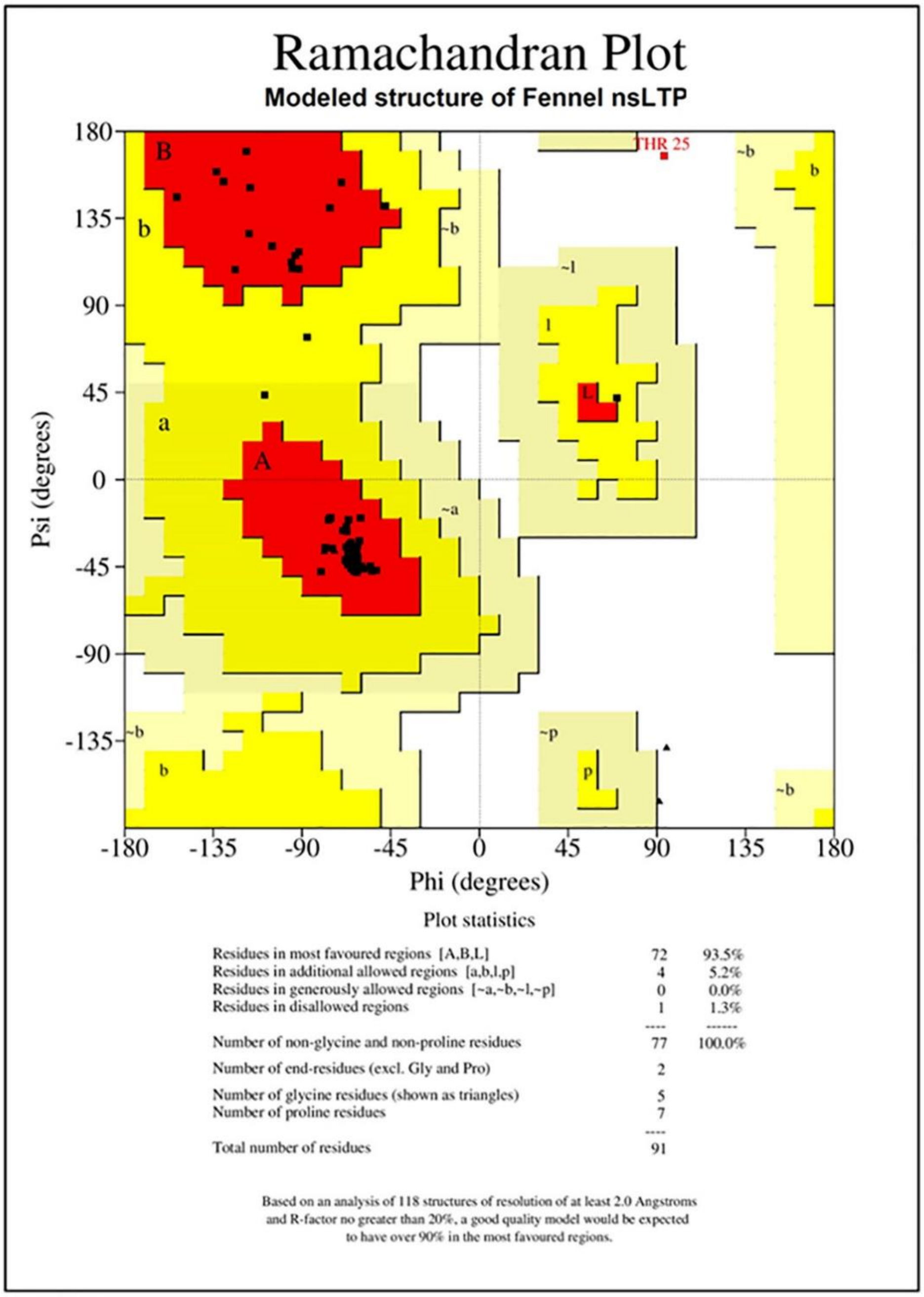

Supplementary Figure S4: Multiple sequence alignment of nsLTP1 from Fennel, Hazelnut (Q9ATH2), Peach (Q9LED1) and Walnut (BMRB 27637) using Clustal Omega. Conserved residues among all sequences are indicated by *. The numbering convention used is according to the Fennel sequence.

```
                                          *         *  *           ** *
Fennel        1 -----------------------AIDCKTVDAALVPCVPYLTGGG-TPTTQCCSGVSSIK
Hazelnut        MGSLKLVCAVLLCMMVAAPVARASLTCPQIKGNLTPCVLYLKNGG-VLPPSCCKGVRAVN
Peach           ------------------------ITCGQVSSSLAPCIPYVRGGG-AVPPACCNGIRNVN
Walnut          -------------------EAEFVITCGQVASSVGSCIGYLRGTVPTVPPSCCNGVKSLN

                    *  *   ** * *             *   * **           ***
Fennel       37 TMAGTPQDKKDACNCVKAAANRYPNIRDDVAQALPVKCNVQLDIPVSRTTNCDAI-
Hazelnut        DASRTTSDRQSACNCLKDTAKGIAGLNPNLAAGLPGKCGVNIPYKISPSTNCNNVK
Peach           NLARTTPDRQAACNCLKQLSASVPGVNPNNAAALPGKCGVSIPYKISASTNCATVK
Walnut          KAAATTADRQAACECLKKTSGSIPGLNPGLAAGLPGKCGVSVPYKISTSTNCKAVK
```

Supplementary Figure S5: Dose-dependent cytotoxic activity on MCF-7 human breast cancer cell line after 48 h treatment. Data from three independent experiments are presented with mean and standard deviation (S.D.) highlighted as *p < 0.05, **P < 0.01, ***P < 0.001. (**a**). Fennel nsLTP1 calculated IC_{50} value 6.98 µM. **(b).** Doxorubicin calculated IC_{50} value 0.037 µM. **(c).** Morphological alteration of MCF-7 cells treated with fennel nsLTP1 for 48 h at different concentrations viewed under phase-contrast microscopy (10x magnification).

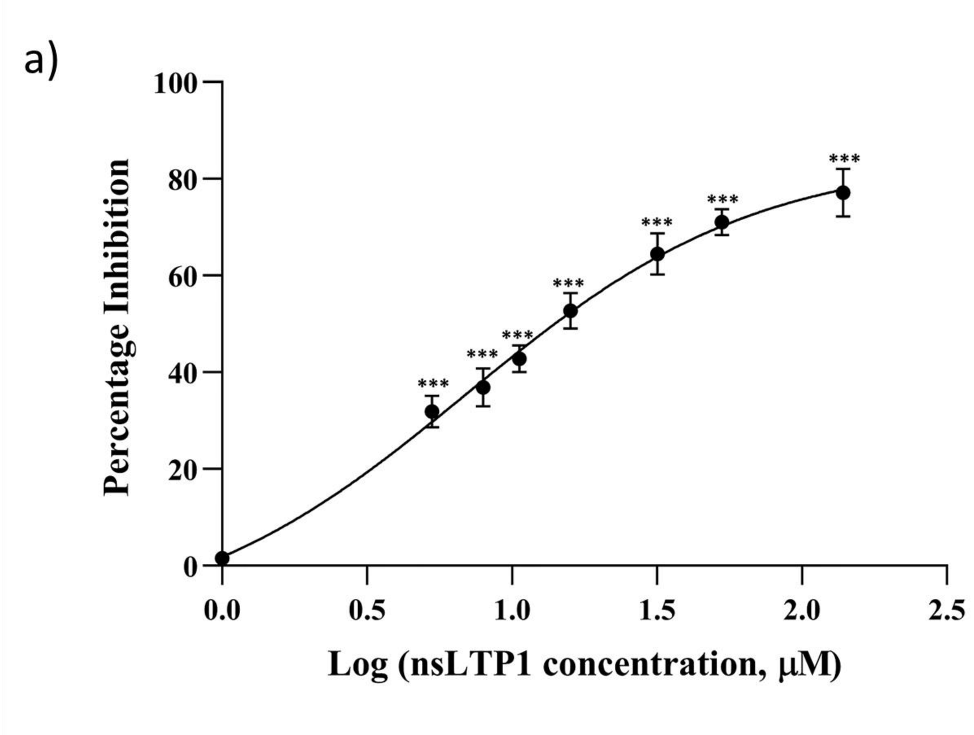

b)

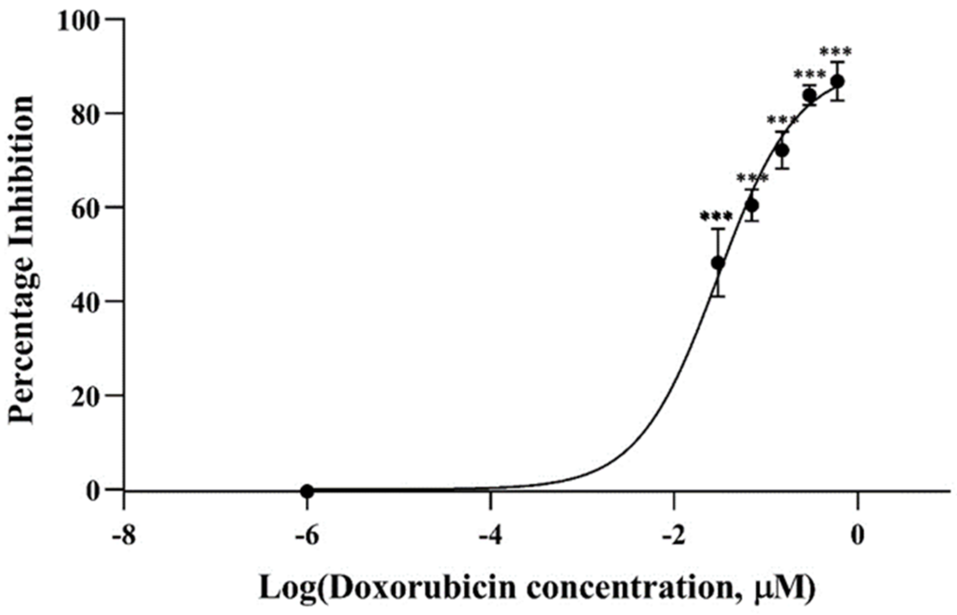

Percentage Inhibition
100
80
60
40
20
0
-8
-6
-4
-2
0
Log(Doxorubicin concentration, μM)
***

c)

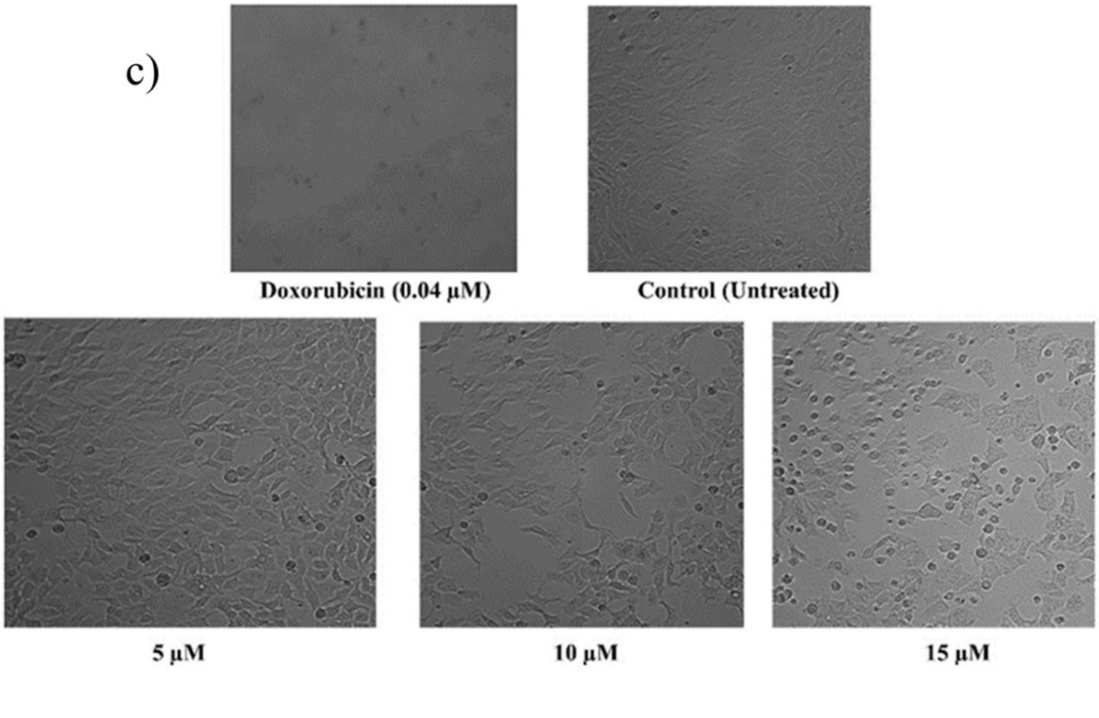

Doxorubicin (0.04 μM)
Control (Untreated)
5 μM
10 μM
15 μM
